# Antimicrobial resistance patterns of *Staphylococcus* species isolated from cats presented at a veterinary academic hospital in South Africa

**DOI:** 10.1186/s12917-017-1204-3

**Published:** 2017-09-15

**Authors:** Daniel Nenene Qekwana, Dikeledi Sebola, James Wabwire Oguttu, Agricola Odoi

**Affiliations:** 1 University of Pretoria, Faculty of Veterinary Science, Department of Paraclinical Sciences, SectionVeterinary Public Health, Pretoria, Gauteng South Africa; 2 University of South Africa, College of Agriculture and Environmental Sciences, Department of Agriculture and Animal Health, Johannesburg, South Africa; 3 University of Tennessee, College of Veterinary Medicine, Department of Biomedical and Diagnostic Sciences, Knoxville, TN USA

**Keywords:** *Staphylococcus* spp., Antimicrobial resistance, Veterinary hospital, Cats, South Africa

## Abstract

**Background:**

Antimicrobial resistance is becoming increasingly important in both human and veterinary medicine. This study investigated the proportion of antimicrobial resistant samples and resistance patterns of *Staphylococcus* isolates from cats presented at a veterinary teaching hospital in South Africa. Records of 216 samples from cats that were submitted to the bacteriology laboratory of the University of Pretoria academic veterinary hospital between 2007 and 2012 were evaluated. Isolates were subjected to antimicrobial susceptibility testing against a panel of 15 drugs using the disc diffusion method. Chi square and Fisher’s exact tests were used to assess simple associations between antimicrobial resistance and age group, sex, breed and specimen type. Additionally, associations between *Staphylococcus* infection and age group, breed, sex and specimen type were assessed using logistic regression.

**Results:**

*Staphylococcus* spp. isolates were identified in 17.6% (38/216) of the samples submitted and 4.6% (10/216) of these were unspeciated. The majority (61.1%,11/18) of the isolates were from skin samples, followed by otitis media (34.5%, 10/29). Coagulase Positive *Staphylococcus* (CoPS) comprised 11.1% (24/216) of the samples of which 7.9% (17/216) were *S. intermedius* group and 3.2% (7/216) were *S. aureus*. Among the Coagulase Negative *Staphylococcus* (CoNS) (1.9%, 4/216), *S. felis* and *S. simulans* each constituted 0.9% (2/216). There was a significant association between *Staphylococcus* spp. infection and specimen type with odds of infection being higher for ear canal and skin compared to urine specimens. There were higher proportions of samples resistant to clindamycin 34.2% (13/25), ampicillin 32.4% (2/26), lincospectin 31.6% (12/26) and penicillin-G 29.0% (11/27). Sixty three percent (24/38) of *Staphylococcus* spp. were resistant to one antimicrobial agent and 15.8% were multidrug resistant (MDR). MDR was more common among *S. aureus* 28.6% (2/7) than *S. intermedius* group isolates 11.8% (2/17). One *S. intermedius* group isolate was resistant to all β-lactam antimicrobial agents tested.

**Conclusion:**

*S. intermedius* group was the most common cause of skin infections and antimicrobial resistance was not wide spread among cats presented at the veterinary academic hospital in South Africa. However, the presence of MDR-*Staphylococcus* spp. and isolates resistant to all β-lactams is of both public health and animal health concern.

## Background

Although *Staphylococcus* are commensals of the skin, mucous membranes, alimentary and urogenital tracts of a diverse group of mammals and birds, they have been implicated in clinical infections of humans and animals [1–3]. Transmission of *Staphylococcus* between animals and humans are known to occur [1, 4]. Cats have been reported as carriers of both Coagulase positive (CoPS) and coagulase negative *Staphylococc*us species (CoNS) [2, 3, 5–7]. However, coagulase positive *Staphylococcus* species infections seem to be more prominent in feline medicine than CoNS infections [1]. Among the CoPS species in cats, *S. pseudintermedius* are the most common followed by *S. aureus* [5, 8]. These infections have been associated with pyoderma, postoperative wound infections and otitis [9]. In addition, *S. felis*, is a cause of urinary tract infections [10].

Although resistance to β-lactam antimicrobials among *Staphylococcus* isolates from cats has been reported [6, 8], other antimicrobial agents such as gentamycin, enrofloxacin and doxycycline have been reported to be effective against *Staphylococcus* infections in cats [5, 11, 12]. However, information on the proportion of antimicrobial resistant isolates and resistance patterns of *Staphylococcus* species in clinical cases of cats in developing economies in general and South Africa in particular is very limited. Therefore, the objective of this study was to investigate the proportion of antimicrobial resistant isolates and resistance patterns among *Staphylococcus* species isolates from cat samples submitted to a veterinary academic hospital in South Africa between 2007 and 2012.

## Methods

### Data collection

Data containing records of cat samples submitted to the University of Pretoria Bacteriology Laboratory at the Veterinary Teaching Hospital in South Africa between January 2007 and December 2012 for routine diagnostic tests were evaluated. The following variables were captured: breed, age, sex, specimen type, staphylococcus species isolated, antimicrobial included in the antimicrobial susceptibility test panel and the susceptibility profile of the isolates.

### Staphylococcus identification and antimicrobial susceptibility testing

Culture of samples was done using sheep blood agar incubated at 37 °C for at least 24 h. All media used were quality controlled using *S. aureus* ATCC 25923. Suspected *Staphylococcus* colonies were identified based on phenotypic characteristics including colony characteristics, catalase, D-mannitol, maltose, deoxyribonuclease (DNase) tests, polymyxin-B and Gram-staining as described by Quinn [13]. *S. intermedius* and *S. delphini* were classified as *S. intermedius* group (SIG) as described by Sasaki et al. [14].

Isolates were subjected to antimicrobial susceptibility testing against a panel of 15 drugs using the disc diffusion method (discs supplied by Oxoid) [15]. Included in the panel were the following drugs: 30 μg amikacin (AK), 30 μg doxycycline (DOX30), 5 μg enrofloxacin (ENR), 10 μg gentamicin (CN), 10 μg penicillin G (P), 25 μg sulpha-trimethroprim (SXT), 30 μg chloramphenicol (C), 30 μg cephalothin/lexin (KF), 30 μg kanamycin (K), 2 μg clindamycin/lincomycin (MY), 100 μg lincospectin (Espectinomycine-lincomycine) (LS100), 5 μg orbifloxacin (OBX5), 20/10 μg synulox (Amoxicillin-Clavulanic acid) (AMC20/10) and 15 μg tylosin (TY). The results, based on the diameter of the inhibition zones, were classified as sensitive, intermediate or resistant in accordance with the Clinical and Laboratory Standards Institute [15]. For the purposes of the study, intermediate susceptibility was considered as susceptible. Multidrug resistance (MDR) was defined as resistance to at least one antimicrobial agent in three or more antimicrobial categories [16].

### Data analysis

All the statistical analyses were performed using SAS 9.4 (SAS Institute Inc., Cary, NC, USA) statistical package. The dataset was assessed for missing data and inconsistencies such as improbable values. Shapiro-Wilk test of normality was used for evaluation of distributions of age that was found to be non-normally distributed and hence median and interquartile ranges were reported. Age was also categorised into two categories: <2 years and ≥2 years. The frequencies and proportions of all categorical variables were calculated and presented in a table. Associations between antimicrobial resistance of *Staphylococcus* spp. isolates and a number of host factors (breed, age, sex, specimen type) and other categorical variables were assessed using the Chi-square and Fisher’s Exact tests. Statistical significant was assessed using a critical *p*-value of 0.05. The variables specimen type and breed had too many categories to include in the model in their original form and hence they were re-coded (Table 1).

**Table 1:** Distribution of *Staphylococcus* isolates among cat specimens tested at an academic veterinary hospital laboratory, 2007–2012

		Number of cats tested		*Staphylococcus* positive	
Variable	Category	Frequency	Percent	Frequency	Percent
Breed (*n* = 212)					
	Domestic Short Hair	132	62.3	24	18.2
	Persian	21	9.9	4	19.1
	Siamese	15	7.1	1	6.7
	Domestic Long Hair	12	5.7	3	25.0
	All others	32	15.1	6	18.8
Sex (*n* = 205)					
	Male	122	59.5	17	13.9
	Female	83	40.5	20	24.1
Specimen type (*n* = 215)					
	Urine	95	44.2	3	3.2
	Ear canal swab	29	13.5	10	34.5
	Skin	18	8.4	11	61.1
	All others	73	34.0	14	19.2
Age (*n* = 216)					
	≥2 years	123	56.9	21	17.1
	<2 years	93	43.1	17	18.3

### Univariable and multivariable models

Investigation of the predictors of *Staphylococcus* spp. infections was done in two steps. In the first step, univariable logistic regression model was fit to assess the relationships between sex, age, specimen type and breed, and the outcome variable, *Staphylococcus* status. The potential predictors of *Staphylococcus* spp. infection at this stage were identified using a relaxed α ≤ 0.20. Thus variables with *p* ≤ 0.20 in the univariable model were considered for inclusion in the multivariable model in the 2nd step. Therefore, the 2nd step involved fitting a multivariable logistic regression model using manual backwards selection method with the significance set at α ≤ 0.05.

Confounding was assessed by comparing the change in model coefficients with and without the suspected confounders. If the removal of a suspected confounding variable resulted in a 20% or greater change in another model coefficient, the removed variable was considered a confounder and retained in the model regardless of its statistical significance. In addition, two-way interaction terms between variable in the final main effects model were assessed.

Odds ratios (ORs) and their 95% confidence intervals were computed for variables included in the final model. The differences between categories of statistically significant predictors for *Staphylococcus* spp. were also assessed by changing the reference categories of the predictors. Hosmer-Lemeshow goodness-of-fit test was used to assess model fit.

## Results

A total of 216 samples were submitted to the bacteriology lab during the study period, of which, 17.6% (38/216) tested positive for *Staphylococcus* spp. each of which had single isolates (i.e. no mixed infections were identified).The majority of samples tested were urine (44.2%, 95/215), followed by ear canal swab (13.5%, 29/215) and skin samples (8.4%, 18/215). Significantly (*p* = 0.0065) more samples originated from males (59.5%, 122/205) than female cats (40.5, 83/205). Similarly, a significantly (*p* = 0.0412) higher proportion of samples came from cats ≥2 years (56.9%, 123/216) compared to cats <2 years (43.1%, 93/216). The majority of samples were obtained from the domestic short hair breed (DSH) (62.3%, 132/212), followed by Persian breed (9.9%, 21/212) (Table 1).

*Staphylococcus* species were isolated from several cat breeds including domestic long hair (25.0% 3/12), domestic short hair (18.2%, 24/132) and persian breed (19.1%, 4/21). Skin samples yielded the highest (61.1%,11/18) percentage of *staphylococcus* isolates followed by ear swabs (34.5%,10/29).

Significantly (*p* = 0.02) more CoPS (11.1%, 24/216) were isolated compared to CoNS (1.9%, 4/216). Among the CoPS, *S. intermedius* group was most predominant (7.9%, 17/216) followed by *S. aureus* (3,2%, 7/216). Equal percentage of *S. felis* (0.9%, 2/216) and *S. simulans* (0.9%; 2/216) were observed among the CoNS. Five percent (4.6%, 10/216) of the *Staphylococcus* isolates identified were not characterized (Table 2).

**Table 2:** Distribution of *Staphylococcus* species isolated from clinical specimens from cats presented at an academic veterinary hospital between 2007 and 2012 (*n* = 216)

	Isolate	Frequency	Percent (%)
CoPS *n* = 24 (11.1%)	*S. intermedius* group (SIG)	17	7.9
	*S. aureus*	7	3.2
CoNS *n* = 4 (1.9%)	*S. felis*	2	0.9
	*S. simulans*	2	0.9
Unspeciated	*S*. spp.	10	4.6
	Negative	172	82.4
	Total	216	100

*Staphylococcus* isolates exhibited relatively high levels of resistance towards ampicillin (32.4%, 12/26), penicillin-G (29.0%, 11/27), clindamycin (34.2%,13/25) and lincospectin (31.6%, 12/26) (Table 3). Overall, 63.2% (24/38) of *Staphylococcus* spp. were resistant to at least one antimicrobial agent and 21.1% (8/38) were multidrug resistant (MDR). *S. aureus* (85.7%, 6/7) had the highest level of resistance to at least one antimicrobial agent followed by *S. intermedius* group (52.9%, 9/17). Similarly, *S. aureus* (42.9%, 3/7) had a higher level of MDR than *S. intermedius* group (12.5%, 2/16). One *S. intermedius* group isolate was resistant to all β-lactam antimicrobial agents tested. This isolate was also resistant to 9 out of the 15 antimicrobial agents tested. Three *S. intermedius* group isolates were resistant to both clindamycin and lincosamides. Among the *S. aureus* isolates*,* one was resistant to five antimicrobial agents and two to four antimicrobial agents (Table 4).

**Table 3:** Antimicrobial resistance profile of *Staphylococcus* isolates to antimicrobial agents from samples tested at an academic veterinary laboratory, 2007–2012

Group	Drug	Frequency	Percent (n/N) ^b^
β-lactam		26	28.9 (26/90)
Penicillin	PenicillinG	11	29.0 (11/27)
	Ampicillin	12	32.4 (12/26)
Cephalosporin	Cephalothin	1	2.7 (1/37)
	Ceftiofur	1	2.7 (1/37)
Combination	Amoxicillin/Clavulanic acid	1	2.7 (1/37)
Tetracycline	Doxycycline	1	2.7 (1/37)
Fluoroquinolone		6	8.6 (6/70)
	Enrofloxacin	3	7.9 (3/35)
	Orbifloxacin	3	7.9 (3/35)
Aminoglycoside		4	3.6 (4/110)
	Gentamicin	1	2.7 (1/37)
	Amikacin	1	2.7 (1/37)
	Kanamycin	2	5.3 (2/36)
Potentiated sulfonamide	Sulfamethoxazole/trimethoprim	4	10.5 (4/34)
Amphenicols	Chloramphenicol	2	5.2 (2/36)
Lincosamides	Clindamycin	13	34.2 (13/25)
Aminoglycoside-lincosamide	Lincospectin	12	31.6 (12/26)
Macrolide	Tylosin	2	5.3 (2/36)

^b^ = n is the number resistant, N is number tested

**Table 4:** Antimicrobial resistance patterns identified in *Staphylococcus* isolates from cat specimens tested at an academic veterinary hospital laboratory, 2007–2012

	Antimicrobial Resistance	Multidrug Resistance	B-Lactam resistance	Resistance patterns
Species	Percent (n/N)	Percent (n/N)	Percent (n/N)	
*S. aureus*	85.7 (6/7)	42.9 (3/7)	0	AMP (1), AMP PEN (1), AMP PEN LIN (1), AMP PEN CLI LIN (1), AMP PEN CHL LIN (1), AMP SP KAN CLI LIN (1)
*S. felis*	0 (0/2)	0	0	
*S. intermedius* group	52.9 (9/17)	11.8 (2/17)	5.9 (1/17)	PEN (1), KAN (1), CLI LIN (3), SP LIN (1), AMP PEN SP (1), ENR LIN OR TYL (1), AMP CEF GEN PEN SP CEF KAN OR SU (1)
*S. simulans*	0 (0/2)	0	0	
*S.* spp.	90.0 (9/10)	30.0 (3/10)	0	CLI (2), LIN (1), CHL CLI (1), AMP PEN (1), CLI LIN OR (1), AMP AMP DOX ENR PEN CLI (1), AMP PEN CLI (1), AMP ENR PEN CLI LIN TYL (1),

*n* number of resistant samples, *N* number of samples tested, *AMP* ampicillin, *CEF* Ceftiofur, *ENR* Enrofloxacin, *GEN* Gentamicin, *PEN* PenicillinG, *SP* Sulpha/Trimethroprim, *CHL* Chloramphenicol, *KAN* Kanamycin, *CLI* Clindamycin/Lincomycin, *AMI* Amikacin, *DOX* Doxycycline, *LIN* Lincospectin, *ORB* Orbifloxacin, *SU* Amoxicillin/Clavulanic acid, *TYL* Tylosin

### Predictors of staphylococcus infections

Based on the univariable logistic model, only sex and specimen type stood out as potential predictors of *Staphylococcus* spp. infection based on a liberal α ≤ 0.20 (Table 5). Thus, only these two variables were assessed in the multivariable model. In the final model only specimen type was significantly associated with staphylococcus species infection based on α ≤ 0.05. The odds of testing positive for *Staphylococcus* spp. infections were significantly higher among ear canal (*p* = 0.0002) and skin samples (*p* < 0.0001) than urine samples (Table 6). However, there was no significant differences in the odds of *Staphylococcus* spp. infection between skin and ear canal samples (Table 7).

**Table 5:** Results of the univariable logistic model showing predictors of *Staphylococcus* spp. infection among cats tested at an academic veterinary hospital laboratory, 2007–2012

Variable	OR^a^	95% CI^b^		p-value
Breed				
Domestic Long Hair	1.4	0.3	7.0	0.368
Domestic Short Hair	0.9	0.4	2.6	0.707
Persian	1.0	0.3	4.2	0.723
Siamese	0.3	0.03	2.8	0.237
All others	Ref	.	.	.
Sex				
Female	1.9	0.9	4.0	0.066
Male	Ref	.	.	.
Specimen type				
Ear canal swab	16.1	4.1	64.2	<0.0001
Skin	48.2	10.9	213.7	<0.0001
All others	7.3	2.0	26.4	0.003
Urine	Ref	.	.	.
Age				
< 2 years	1.1	0.5	2.2	0.818
> = 2 years	Ref	.	.	.

^a^Odds ratio

^b^95% Confidence Interval

**Table 6:** Multivariable logistic model showing predictors of *Staphylococcus* spp. infection among cats tested at an academic veterinary hospital laboratory, 2007–2012

Variable	OR^1^	95% CI^2^		p-value
Sex				
Female	1.9	0.8	4.3	0.117
Male	Ref	.	.	.
Specimen type				
Ear canal swab	14.8	3.6	60.5	0.0002
Skin	52.1	11.3	240.3	<.0001
All others	8.4	2.3	30.7	0.001
Urine	Ref	.	.	.

**Table 7:** Final multivariable logistic model showing the results of changing reference categories of specimen type

Variable	OR^1^	95% CI^2^		p-value
Specimen type				
Skin	3.519	0.969	12.78	0.0559
Urine	0.068	0.017	0.276	0.0002
All others	0.564	0.208	1.531	0.2611
Ear canal swab	Ref	.	.	.
Ear canal swab	0.284	0.078	1.032	0.0559
Urine	0.019	0.004	0.089	<.0001
All others	0.16	0.05	0.517	0.0022
Skin	Ref	.	.	.
Ear canal swab	1.773	0.653	4.81	0.2611
Skin	6.237	1.933	20.131	0.0022
Urine	0.12	0.033	0.439	0.0014
All others	Ref	.	.	.

## Discussion

The aim of this study was to investigate the proportion of antimicrobial resistant isolates and resistance patterns of *Staphylococcus* spp. isolates from clinical samples obtained from cats admitted to a veterinary academic hospital in South Africa. The proportion of *Staphylococcus* spp. isolated from cat samples in this study was relatively low (17.6%). This is not directly comparable to findings from other previous studies on cats due to differences in isolation methods (use of enrichment media in particular), and differences in study designs. In the current study, we investigated *Staphylococcus* infections in hospitalised clinical cases only. However, the majority of similar published studies of *Staphylococcus* in cats have largely focused on methicillin resistance rather than *Staphylococcus* infections in general. In addition, past studies have focused on carriage rather than infections [8, 17].

Similar to findings from other studies [8, 17], in this study we observed that skin and ear canal samples had significantly higher odds of testing positive for *Staphylococcus* spp. than other samples. These results seem to suggest that *Staphylococcus* spp. are a major cause of skin related infections in cats [18–20]. Although there tended to be a higher proportion of *Staphylococcus* spp. isolated from the domestic short hair breeds, the final model indicated no significant association between breed and odds of *Staphylococcus* spp. infection. However, the lack of significant association might be due to small sample size involved in this study. It is worth noting that, there is evidence that certain diseases are more common in certain breeds of cats and we suspect that this might be the case with *Staphylococcus* infections [17, 21].

Consistent with other studies [3, 5, 17, 19], we observed a higher percentage of CoPS than CoNS. This is mainly due to the observed higher percentage of *S. intermedius* group, which are CoPS, isolated in this study. On the contrary, Abraham et al. [7] reported nearly equal proportions of *S. aureus* and *S. pseudintermedius* isolates from asymptomatic cats. Consistent with our study, a Brazilian study by Lilenbaum et al. [8] reported a higher percentage of *S. intermedius* group (*S. pseudintermedius*) in cats compared to other *Staphylococcus* species. This may be related to the fact that SIG especially *S. pseudintermedius* is well adapted to the skin surface of dogs and cats than *S. aureus* [22–25].

The observed higher percentage of resistance towards β-lactam and lincosamide antimicrobial agents among the *Staphylococcus* isolates in cats has previously been reported [6, 8, 23]. Of particular concern is one *S. intermedius* group isolate that was resistant to all β-lactam antimicrobial agents tested in this study. Moreover, MRSA have an intrinsic resistance to β-lactams by virtue of newly acquired low-affinity penicillin-binding protein 2A (PBP2A). Therefore, it is possible that this isolate was MRSA [26, 27]. Unforunately, we could not assess this since the lab that supplied the data used in this study did not test for methicillin resistance. Almost 16 % (15.8%) of *Staphylococcus* isolates in this study were MDR. This is close to the 14.8% reported by Gandolfi-Decristophoris et al. [23] in Switzerland.

Since this is a retrospective study, these findings should be interpreted with caution. The history of previous use of antimicrobial agents was not included in the analysis and this could have affected the recovery rates of *Staphylococcus* species. The study also suffers from low samples size which impacted the precision of some of the estimates. Nonetheless, the results provide a useful preliminary indication of the burden and antimicrobial resistance patterns of *Staphylococcus* spp. infections in cats presented to the academic veterinary hospital in South Africa.

## Conclusions

As has been observed in other studies, this study suggests that *S. intermedius* group is the most common cause of skin infections in cats investigated in this study. It also suggests that antimicrobial resistance is not so wide spread among cats presented at the veterinary academic hospital in South Africa. Considering the risk of cross-transmission of resistant organisms between cats and humans, the levels of resistance to β-lactams is of great concern from both a public health and animal health point of view. However, given the limited scope of this study, there is need for larger and more detailed primary base studies to specifically assess the extent of antimicrobial resistant infections in cats in South Africa and their role in the spread of antimicrobial drug resistance to humans.

## Abbreviations

AMR: Antimicrobial resistance
CoNS: Coagulase negative staphylococci species
CoPS: Coagulase Positive *Staphylococcus*
DSH: Domestic Short Hair breed
MDR: Multidrug resistant resistance
SAS: Statistical Analysis System
